# An Overview of Diabetes Mellitus in Egypt and the Significance of Integrating Preventive Cardiology in Diabetes Management

**DOI:** 10.7759/cureus.27066

**Published:** 2022-07-20

**Authors:** Mohamed R Abouzid, Karim Ali, Ibrahim Elkhawas, Shorouk M Elshafei

**Affiliations:** 1 Internal Medicine, Baptist Hospitals of Southeast Texas, Beaumont, USA; 2 Stem Cell and Cancer Biology, Mayo Clinic, Rochester, USA; 3 Internal Medicine, Steward Carney, Dorchester, USA; 4 Internal Medicine, Mansoura University Faculty of Medicine, Mansoura, EGY

**Keywords:** coronary artery calcium score, preventive cardiology, pesticides, smoking, obesity, hepatitis c virus, diabetes epidemiology, diabetes in egypt

## Abstract

In Egypt, diabetes mellitus (DM) is a significant public health concern, and the disease is considered a modern pandemic throughout the world. The incidence of diabetes is steadily climbing, which is causing grave concern. As a result, it is essential to take into consideration the risk factors that are pervasive in Egyptian society and have led to the worsening of this problem. These risk factors include sedentary lifestyles, obesity, hepatitis C infections, pesticides, smoking, and bad cultural habits. In this review, we aim to demonstrate the possible solutions to fight diabetes mellitus and overcome its serious health and socioeconomic burdens in Egypt. A multidisciplinary, team-based approach is highly recommended in diabetes management. Primary care physicians, endocrinologists, nephrologists, and preventive cardiologists all play a crucial role in providing the highest possible level of care to diabetic patients by collaborating closely with one another. The assessment of cardiovascular risk and the prevention of life-threatening cardiovascular events, common among diabetic patients, warrant the introduction of preventive cardiology, a new and significant concept in diabetes care that demands adoption. The integration of preventive cardiology into the treatment of diabetic patients is expected to significantly cut down the morbidity and mortality rates associated with diabetes mellitus and provide them with a better quality of life.

## Introduction and background

Diabetes mellitus (DM) is one of the top ten major causes of death globally. The incidence and prevalence of diabetes mellitus is on the rise, posing a considerable health and economic burden [1]. The increased incidence has reached epidemic proportions. DM is defined as a chronic condition of carbohydrate metabolism caused by insulin insufficiency or failure to respond to insulin, resulting in increased blood glucose. There are three primary types: type 1 DM, type 2 DM (the most common), and gestational DM. Long-term uncontrolled DM may result in micro-and macro-vascular complications that result in morbidity and mortality in diabetic individuals [2].

Egypt is a transcontinental nation that is located in the Mediterranean, North Africa, and the Middle East. Egypt has one of the longest and most significant histories, as proven by the early developments in agriculture, urbanism, writing, construction, religion, and even medicine that were observed and written in ancient Egypt. The ancient Egyptians had extensive knowledge of the human body and medicine thousands of years ago. Interestingly, medical specialization is not a new notion. Physicians in ancient Egypt specialized in specific bodily parts, such as the head, teeth, eyes, and abdomen [3].

Regarding the discovery of DM, we must remember the ancient Egyptian physician Hesy-Ra, who was the first to hint at its existence in 1552 BC. He recorded his account of the symptoms of diabetes on an Egyptian Papyrus [4].

In Egypt, DM is a dilemma and a fast-expanding concern. According to UN estimates, Egypt's population is projected to reach 102,334,404 by mid-2020. According to the International Diabetes Federation (IDF), the prevalence of DM among Egyptian adults is 15.2%, which may be an underestimation. Therefore, DM should be thoroughly explored in terms of its risk factors, prevention, treatment, and consequences. Moreover, the general population should be aware of and well informed about all aspects of diabetes [3,4].

## Review

Diabetes epidemiology in Egypt

Data regarding the epidemiology of DM in Egypt are sparse. Nevertheless, according to the IDF, Egypt ranks ninth in the prevalence of DM worldwide, and the number of adult diabetic patients was 8,850,400 in early 2020, with a prevalence of 15.2%. Egypt is a member of the Middle East and North Africa (MENA) region of the IDF [2]. IDF estimates that the number of diabetic patients in the MENA region would double by 2045, reaching 108 million. In actuality, 40% to 50% of people with diabetes or prediabetes go undiagnosed, despite the fact that these numbers may appear to be quite high. The number of diabetic patients in Egypt is expected to be reached by 2035 [2,3]. In Egypt, DM is the leading cause of chronic kidney failure, blindness, amputation of the lower extremities, stroke, and acute coronary syndrome [4].

The economic impact of DM in Egypt

Diabetes is generally acknowledged as a significant socioeconomic burden. The costs associated with diabetes fall into three categories [3,4]. (i) Direct indicates the cost of direct medical care, health care facilities, and hospitals [4]. (ii) Indirect refers to the time lost owing to missing workdays (absenteeism), decreased productivity at work (presentism), decreased labor participation due to disability, and productivity losses due to early retirement and mortality [4]. (iii) Intangible refers to the accompanying changes in the quality of life of patients and family members as a consequence of DM, such as the cost of pain, bereavement, and suffering to individuals and families [4]. The cost of diabetes in the Middle East was projected to be $13.6 billion in 2013 (14% of its total health care costs). Annual cost analysts indicated in 2010 that the economic loss due to type 2 diabetes in Egypt is $1.29 billion per year (regardless of the cost associated with prediabetes and reduced productivity). Egypt has the lowest diabetes-related expenses ($116 per patient per year) among the MENA-region nations. The average annual expenditure per patient in other MENA-region nations ranges from $160 to $3000. This is even less than the $2000 to $7000 per patient per year found in developed nations [5].

The most prevalent risk factors in Egypt

Obesity

Obesity is the most common cause of diabetes. Adipose tissue in obesity, particularly central obesity, releases non-esterified fatty acids, pro-inflammatory cytokines, glycerol, and hormones that contribute to the development of insulin resistance via various biochemical processes. Egypt has one of the highest rates of obesity in the world, particularly among women, which has a negative impact on individual health and costs the state a lot of money in terms of medication and operations to treat the consequences of obesity [4,5]. After Saudi Arabia and the United Arab Emirates, Egypt has the third-highest obesity prevalence in the MENA region. Obesity prevalence in Egypt is somewhat comparable to that of Native American and Hispanic populations. According to the Egypt demographic and health census from 2008, around 50% of Egyptian men and 65-80% of Egyptian women are overweight or obese [5]. According to a World Health Organization (WHO) report from 2010, 30.3% of Egyptians are obese. Obesity is a leading cause of cardiovascular disease, diabetes, and osteoarthritis in Egypt. The most prevalent risk factors for obesity include inherited, which cannot be changed, as well as poor eating habits and physical inactivity, which may be modified [3-5].

Lack of Physical Exercise/Sedentary Lifestyle

Physical inactivity was reported in 81% of the 4918 Cairo households assessed in 1995 [6]. The primary causes of these findings are a lack of awareness and sufficient education about the need for exercise, as well as restricted exercise facilities that are not accessible to everyone, particularly in rural areas. Due to the overcrowding of people and traffic, Egyptians avoid walking or running in public places, which may be their only alternative due to limited and expensive access to gyms or sports clubs. Vitamin D deficiency is common in Egypt due to a lack of sun exposure, and it has been linked to obesity and diabetes [7-10].

Hepatitis C Infection

Egypt has the world's highest incidence of chronic hepatitis C infection. This is a result of the major bilharzia therapy campaigns conducted between 1960 and 1980 with intravenous medicines and non-sterile needles [4]. This incidence resulted in the spread of illness among Egyptians [4].

According to the Egypt Demographic and Health Survey, roughly 15% of Egyptians are serologically positive for hepatitis C virus (HCV) antibodies and 10% have active infection [11]. Diabetes type 2 is frequent in patients with chronic hepatitis C infection. A study was conducted on 9,841 individuals older than 20 years old for whom data on HCV infection and diabetes were collected. It was discovered that 8.4% had type 2 diabetes and 2.1% were positive for anti-HCV antibodies [12]. Chronic hepatitis C infection raises the likelihood of acquiring diabetes complications. A study of 438 patients with type 2 DM (113 Egyptians and 325 Kuwaitis) revealed that those with hepatitis C infection had the poorest glycemic control [13]. In another cross-sectional research of 489 Egyptian individuals with type 2 DM attending an outpatient clinic and dialysis unit, the prevalence of HCV infection was 12.9% among outpatient clinic patients and 18.7% among dialysis unit patients [14]. In pre-diabetic patients, eradication and early treatment of hepatitis C infection can prevent the development of type 2 diabetes, improve glycemic management, and minimize the likelihood of progression to type 2 diabetes.

Pesticides

DM is a multifactorial illness with hereditary and environmental factors. The risk factor status of environmental chemicals cannot be overlooked. A potential risk factor for developing type 2 DM is exposure to agriculture-related pesticides. Egypt is the fifth most pesticide-using country in Africa, hence the association between pesticides and DM must be taken into account in Egypt [15-17]. There is a correlation between type 2 DM and exposure to organ chlorine, DDT, and heptachlor, according to a systematic review and meta-analysis of 22 observational studies examining the relationship between pesticides and type 2 DM [18]. A second population-based case-control research was done among Thailand farmers, including 866 cases with DM and 1021 healthy controls, with the additional DM risk variables accounted for. It has been established that exposure to pesticides is significantly associated with the prevalence of diabetes [19]. It has been hypothesized that pesticides can interfere with normal pancreas function, reduce insulin secretion, or damage mitochondrial cells [20]. There are two ways to be exposed to pesticides; the direct route typically affects farmers, but the indirect way affects the majority of Egyptians due to their exposure to low levels of pesticides in contaminated food. Dichlorodiphenyltrichloroethane (DDT), which is an organic chlorine compound, Malathion, which is an organic phosphorus compound, and chlorpyrifos, which is an organic phosphorus compound, are the most widely used pesticides in Egypt, and the high prevalence of DM in Egypt in recent years may be attributed to the excessive use of these pesticides in agriculture [21-24].

Smoking

Tobacco smoking is a risk factor for type 2 DM. According to the 2014 Surgeon General's Report, smokers have a substantially higher risk of getting type 2 DM than nonsmokers, and this risk increases as the number of cigarettes smoked per day increases. Furthermore, smoking lowers the responsiveness to anti-diabetes medications and increases the chance of developing diabetic complications such as end-stage renal disease, leg ulceration, amputation, peripheral neuropathy, retinopathy, blindness, coronary artery disease, and stroke [25-32]. The underlying mechanism is that smoking and its chemicals cause an inflammatory response in the body. This inflammation produces cell damage and swelling, interfering with normal cell function. Furthermore, smoking causes oxidative stress, which occurs when chemicals in cigarette smoke interact with oxygen in the body, forming oxygen-free radicals that cause tissue damage [33].

Another mechanism is that smoking has been linked to central obesity, which is a risk factor for DM. It was discovered that smokers have higher serum cortisol levels than nonsmokers. Cortisol plays a major role in central obesity and diabetes. Despite the high cost of cigarettes, the proven hazards of smoking, and increased public health education, cigarette smoking remains a severe public health problem in Egypt. Data from a study recently disclosed by the Central Agency for Mobilization and Statistics, according to the 2018 population census data, demonstrate that the number of smokers in Egypt is approximately 11 million Egyptians over the age of 15. Male smokers outnumbered females by 34.2% to 0.2 %. According to this survey, Egypt has over 30 million passive smokers.

Cultural factors predisposing to DM

Bad Dietary Habits

Egyptians tend to follow the Mediterranean diet, which consists primarily of vegetables, legumes, fruits, and fish, with moderate amounts of animal protein. Egyptians, on the other hand, consume significant amounts of white bread and polished rice, both of which have a high carbohydrate content and a high glycemic index [7]. Furthermore, Egypt is one of the world's largest users of trans fat. Trans fat promotes dyslipidemia by raising low density lipoprotein (LDL) cholesterol while decreasing high density lipoprotein (HDL) cholesterol, increasing the risk of type 2 diabetes. Trans-fat can be present in many of the items that Egyptians consume in high quantities, including margarine, cakes, cookies, biscuits, and fried dishes. Furthermore, junk food is an extremely harmful phenomenon that is prevalent in cities [4]. Junk food is heavy in calories, salt, and fat but low in nutritional value, contributing to central obesity and type 2 DM. Nonetheless, in rural areas with a higher percentage of poverty, the diet consists primarily of items with high carbohydrate and fat content and fewer animal proteins. If the aforementioned unfavorable eating habits are followed on a regular basis for an extended period of time, they will undoubtedly result in the development of type 2 DM in vulnerable individuals [3,7].

Decreased Level of Health Awareness

Numerous diabetes patients, particularly those who are uneducated and reside in rural, impoverished areas, are unaware of the dangers and consequences of obesity and DM. They are unaware of the significance of continuous glucose monitoring as well as eye and foot examinations for screening and early detection of complications. In Egypt, there is no check-up policy or wellness check. Dietary restriction, weight loss, and exercise are the initial measures in diabetes care prior to the initiation of medication. However, the vast majority of patients do not adhere to these instructions and are unwilling to alter their lifestyle and diet. Even with prescriptions, noncompliance with anti-diabetic meds is prevalent in Egypt in the form of skipping doses, overdosing, or taking chemicals or herbs provided by a friend or someone other than a physician. Except in a very small number of facilities, government hospitals do not have a policy for the routine monitoring and evaluation of diabetic patients in terms of their hemoglobin A1c level, eye, foot, or kidney function. Patients visit government hospitals only to request medication for free or at a reduced cost, while the majority of patients go to private clinics, where they can receive better health services and care than in government hospitals, but at a higher price. Due to the high cost of private practice, patients rarely get routine examinations or evaluations. All of these issues make glycemic management in Egypt a significant challenge.

Role of preventive cardiologists in diabetes care

Cardiovascular disease is the leading cause of morbidity and mortality in diabetics [34]. Diabetes significantly increases the risk of atherosclerosis, acute coronary syndrome, and leg amputation [35]. Therefore, preventive cardiologists, along with primary care physicians and endocrinologists, should be included in the management of diabetic patients. Preventive cardiology is a medical specialty concerned with maintaining cardiovascular health and preventing the occurrence or recurrence of cardiovascular problems. Given their high cardiovascular risk, this form of therapy is strongly advised for diabetic patients in order to reduce their risk of hospitalization and mortality from severe cardiac events. The purpose of preventive cardiology is to reduce the patient's risk of developing or worsening a heart problem using a combination of lifestyle education, medical therapy, and comprehensive risk assessment [36,37].

Preventive cardiologists now have more tools than ever before to assist them in assessing and reducing their patients' cardiovascular risk [38]. Coronary artery calcium (CAC) scoring has evolved as a widely available, cost-effective, and reproducible tool for measuring the risk of major cardiovascular events like atherosclerosis and coronary artery disease [39]. CAC scoring is particularly beneficial in asymptomatic patients for guiding the use of primary preventive measures such as aspirin and statins [39,40]. In addition, there has been a flood of new data in recent years demonstrating the cardiovascular benefits of newer diabetic medicines, particularly sodium-glucose co-transporter 2 (SGLT2) inhibitors and glucagon-like peptide 1 (GLP-1) receptor agonists, with the FDA subsequently emphasizing cardiovascular indications for many of these medications [41,42]. However, preventive cardiology is a relatively new concept in Egypt, and the ministry of health and relevant authorities should strongly explore its application to the care protocol for diabetic patients.

Solutions for better handling of DM problem In Egypt

Better solutions for the handling of DM problem in Egypt are as follows: (i) Through public health insurance, the ministry of health should implement a screening and follow-up program for diabetic patients in government and university hospitals along with providing glucose monitoring devices at an affordable price. (ii) Raising public awareness of healthy lifestyles, obesity control, diabetes prevention, and adequate nutrition through proper health education in schools, universities, and the media. (iii) Physicians should talk with patients in an empathetic manner, encourage them to freely share their concerns, involve them in the management plan, and build a comfortable DM management program that is appropriate for each patient and helps them to comply with it. (iv) This metabolic epidemic should be fought with the inclusion of qualified diabetes educators and dieticians, preventive cardiologists, and well-trained primary care physicians. (v) Hepatitis C infection should be detected and treated at an early stage. (vi) Pesticide usage regulation in agriculture, provision of protective equipment for farmers and other individuals in direct contact with pesticides, pesticide residue monitoring in food and water, and additionally, education regarding the safe management of these pesticides and how to avoid their dangers are required. (vii) The government should increase the number of seats for endocrinology training and fellowship to overcome the shortage of endocrinologists. (viii) The ministry of health and relevant authorities should offer high-quality subspecialty training in preventive cardiology and include preventive cardiology clinics in hospitals.

## Conclusions

Globally, DM is regarded as a modern pandemic, and it is a significant public health concern in Egypt. The prevalence of diabetes is escalating to alarming levels. Therefore, it is important to consider the risk factors that are prevalent in Egyptian society and have contributed to this escalating issue. Obesity, sedentary lifestyle, hepatitis C infection, pesticides, smoking, and poor eating habits are the primary causes of the rapidly increasing prevalence of DM, which necessitates effective strategies for DM care as well as collaboration between individuals, physicians, and the government. Primary care physicians, endocrinologists, and preventive cardiologists are integral elements who work synergistically to ensure the best care for diabetic patients. Preventive cardiology is a new, significant concept in diabetes care that merits implementation for the assessment of cardiovascular risk and prevention of life-threatening cardiovascular events that are common among diabetic patients. The involvement of preventive cardiology in the management of diabetic patients can remarkably reduce the morbidity and mortality associated with DM.
